# Medial Orbitofrontal Cortex Is Associated with Fatigue Sensation

**DOI:** 10.1155/2010/671421

**Published:** 2010-06-10

**Authors:** Seiki Tajima, Shigeyuki Yamamoto, Masaaki Tanaka, Yosky Kataoka, Masao Iwase, Etsuji Yoshikawa, Hiroyuki Okada, Hirotaka Onoe, Hideo Tsukada, Hirohiko Kuratsune, Yasuomi Ouchi, Yasuyoshi Watanabe

**Affiliations:** ^1^Hyogo Children's Sleep and Development Medical Research Center, Hyogo Rehabilitation Centre Central Hospital, 1070 Akebono-cho, Nishi-ku, Kobe 651-2181, Japan; ^2^Research Center for Child Mental Development, Hamamatsu University School of Medicine, 1-20-1 Handayama, Hamamatsu, Shizuoka 431-3192, Japan; ^3^Department of Physiology, Osaka City University Graduate School of Medicine, 1-4-3 Asahimachi, Abeno-ku, Osaka 545-8585, Japan; ^4^Center for Molecular Imaging Science, RIKEN Kobe Institute, 6-7 Minatoshima-minamimachi, Chuo-ku, Kobe, Hyogo 650-0047, Japan; ^5^Department of Psychiatry, Osaka University Graduate School of Medicine, 2-2 Yamadaoka, Suita, Osaka 565-0871, Japan; ^6^Central Research Laboratory, Hamamatsu Photonics, 5000 Hirakuchi, Hamamatsu, Shizuoka 434-8601, Japan; ^7^Department of Health Sciences, Faculty of Health Sciences for Welfare, Kansai University of Welfare Sciences, 3-11-1 Asahigaoka, Kashihara, Osaka 582-0026, Japan; ^8^Laboratory of Human Imaging Research, Molecular Imaging Frontier Research Center, Hamamatsu University School of Medicine, 1-20-1 Handayama, Higashi-ku, Hamamatsu 431-3192, Japan

## Abstract

Fatigue is an indispensable bioalarm to avoid exhaustive state caused by overwork or stresses. It is necessary to elucidate the neural mechanism of fatigue sensation for managing fatigue properly. We performed H_2_^ 15^O positron emission tomography scans to indicate neural activations while subjects were performing 35-min fatigue-inducing task trials twice. During the positron emission tomography experiment, subjects performed advanced trail-making tests, touching the target circles in sequence located on the display of a touch-panel screen. In order to identify the brain regions associated with fatigue sensation, correlation analysis was performed using statistical parametric mapping method. The brain region exhibiting a positive correlation in activity with subjective sensation of fatigue, measured immediately after each positron emission tomography scan, was located in medial orbitofrontal cortex (Brodmann's area 10/11). Hence, the medial orbitofrontal cortex is a brain region associated with mental fatigue sensation. Our findings provide a new perspective on the neural basis of fatigue.

## 1. Introduction

Fatigue sensation is an indispensable bio-alarm to avoid the exhaustive state caused by overwork or stresses, which may also induce a variety of diseases. Therefore, to avoid the exhaustive state, it is important to understand the neural mechanism of fatigue sensation. In addition, to develop the quantification methods of fatigue sensation, which would also contribute to the establishment of the better treatment methods of fatigue, to know the neural mechanism of fatigue sensation may be useful.

Fatigue can be classified into physical and mental types. Most people experienced more mental fatigue than physical one in modern society. Therefore, we focused on mental fatigue-related sensation in this study. Mental fatigue represents a failure to complete mental tasks in the absence of demonstrable cognitive failure or motor weakness [1]. It is necessary to elucidate neural mechanism of fatigue sensation for managing fatigue properly. However, although neural mechanism of physical fatigue has been to some extent clarified [2–6], little is known concerning the neural substrates of mental fatigue sensation.

Limitations of the previous studies to identify the brain regions associated with fatigue had included that the fatigue scores had been obtained only at baseline, prior to the acquisition of functional brain data. As proposed by Chaudhuri and Behan [7], dissociation between the level of internal input and that of the perceived signal associated with applied voluntary effort may result in the sense of fatigue. Therefore, in order to determine the brain region, neural assessment of the transient task-induced activation rather than task-independent baseline state may be useful. Cook and colleagues performed a functional magnetic resonance imaging (fMRI) study to determine the brain region associated with fatigue sensation during a fatigue-inducing cognitive task in patients with chronic fatigue syndrome (CFS) [8]. Significant positive relationships were found between subjective fatigue sensation and responsiveness in the frontal, temporal, cingulate, and cerebellar regions. It was reported that patients with CFS showed increased fMRI activation in the occipitoparietal cortex, posterior cingulate gyrus and parahippocampal gyrus, and decreased activation in the dorsolateral and dorsomedial prefrontal cortices during provocation of fatigue [9]. Although these findings are useful to identify the brain regions related to fatigue sensation, these were limited to just a specific disorder. In addition, simultaneous evaluation of subjective fatigue sensation was not performed during the fatigue-inducing task in those fMRI studies. We performed H_2_^15^O positron emission tomography (PET) scans for the measurement of adjusted regional cerebral blood flow (rCBF) as an indicator of neural activation concomitant with simultaneous evaluation of fatigue sensation while subjects were performing fatigue-inducing task trials, in order to determine the brain region associated with fatigue sensation in healthy subjects.

## 2. Materials and Methods

### 2.1. Subjects

Twelve right-handed, male healthy volunteers (age, 22.4 ± 4.1 years; mean ± SD) participated in this study. Handedness was assessed according to the Edinburgh handedness inventory [10]. None had a history of psychiatric or neurological illness, and none were taking medications known to affect cerebral blood flow. A psychiatrist (M. I.) carefully interviewed all the subjects in order to exclude potential neuropsychiatric diseases. This study was approved by the Ethics Committee of Hamamatsu Medical Center, and all participants provided written informed consent for participation.

### 2.2. Experimental Design

**Figure 1:**
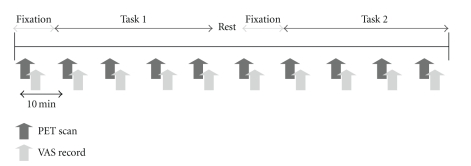
Experimental design. The experiment included 2 sessions. During the experiment, subjects performed 2 types of mental tasks involving advanced trail-making tests (ATMT). During the first session, subjects performed Task 1 of ATMT while during the second session they performed Task 2. At the end of each positron emission tomography (PET) scan, subjects were asked to rate their subjective fatigue sensation on a visual analogue scale (VAS) from 0 (no fatigue) to 100 (total exhaustion). Each session included 10-min fixation and 35-min fatigue-inducing task trails. The session, which included 1 fixation scan and 4 task scans, was performed with 10-min interval. The rest period between the sessions was 8 minutes.

The PET experiment included 2 sessions (Figure 1). During the PET experiment, subjects performed 2 types of mental tasks, which were advanced trail-making tests (ATMTs) [11]. In order to identify the brain regions associated with mental fatigue sensation by using PET technique, it was very important to induce fatigue by performing task trials after relatively short period. The ATMT was chosen as the mentally fatigue-inducing task based on the results of our preliminary studies and a report by Kajimoto [11]. In the ATMT, circles numbered from 11 to 35 were first randomly located on the display of a touch-panel screen. The subjects were required to touch these circles in sequence, starting with circle number 11. When they touched the first target circle, it disappeared and circle number 36 appeared in a different position on the screen. In Task 1 of ATMT, the positions of the other circles remained the same; in contrast, in Task 2, the positions of all the circles were changed. During the first session, subjects performed Task 1 of ATMT, while during the second session they performed Task 2. When they touched the circle numbered 99, the test was ended and then started again without any time intervening. They were instructed to perform these tasks as quickly and correctly as possible. The mean reaction time to touch the ATMT circles during the fatigue-inducing task trials was recorded in order to assess the task performance. Each session included 10-min fixation and 35-min fatigue-inducing task trails. The session, which included 1 fixation scan and 4 task scans, was performed with 10-min interval. During the fixation time, circles numbered from 1 to 25 were randomly located on the screen, and subjects were asked to fix their eyes on the circle number 1 placed at the center of the screen. Immediately after the end of each PET scan, subjects were asked to rate their subjective fatigue sensation on a visual analogue scale (VAS) from 0 (no fatigue) to 100 (total exhaustion) [12, 13], while subjects were performing task trials. In order to minimize interindividual variability in fatigue sensation, standardized VAS score was calculated as (VAS value − mean of VAS value)/(SD of VAS value). The time interval between sessions was 8 minutes.

### 2.3. PET Scan Acquisition

Just before PET measurement, subjects underwent magnetic resonance imaging (MRI) using a 0.3 T scanner (MRP7000AD, Hitachi, Tokyo, Japan). PET scan was performed with a spatial resolution of 3.5 mm at full width at half maximum (FWHM) transaxially and 4.2 mm axially, using as SHR12000 scanner (Hamamatsu Photonics KK, Hamamatsu, Japan) in 3D. Each subject received 370 MBq of H_2_^15^O intravenously through the left antecubital vein for each scan for the measurement of adjusted rCBF.

### 2.4. PET Data Analysis

Statistical parametric mapping software (SPM99; Wellcome Department of Cognitive Neurology, Institute of Neurology, London, UK) was used for image realignment, normalization, and smoothing and to create statistical maps of significant rCBF changes [14, 15]. After realignment, all scan images were stereotaxically normalized to the template of the Montreal Neurological Institute [16] using nonlinear transformation, and the normalized images were smoothed using a Gaussian filter of 7 mm FWHM and the values of adjusted rCBF expressed as ml/100 g/min. Correlation analysis was performed according to the general linear model at each voxel (voxel size: 2.0 × 2.0 × 2.0  mm). The intensity threshold applied to cluster-level statistics was set at a *P*-value less than .001, and the extent threshold in terms of the number of voxels was more than 50.

## 3. Results

**Figure 2:**
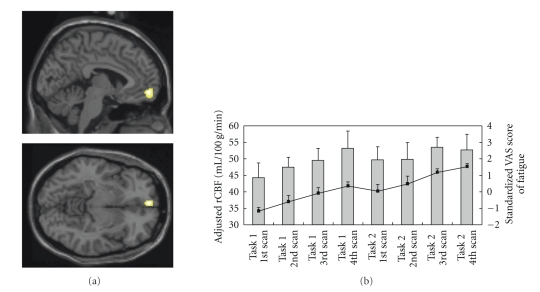
Brain region associated with subjective sensation of fatigue. (a) Sagittal (upper) and horizontal (lower) views of statistical parametric maps superimposed on template brains. Adjusted regional cerebral blood flow (rCBF) in medial orbitofrontal cortex (Brodmann's area 10/11) was significantly correlated with standardized visual analogue scale (VAS) score for fatigue (*P* < .001, Z-score = 4.65, uncorrected). The cluster consisted of 153 voxels extending from the stereotaxic Talairach coordinates (*x* = 14, *y* = 54, and *z* = −9 ). (b) Adjusted rCBF in left medial orbitofrontal cortex and standardized VAS score for fatigue during the task trials. The bar graphs show adjusted rCBF, and the line graphs show standardized VAS score for fatigue, respectively (mean and SD).

The brain region exhibiting a positive correlation in activity with the standardized VAS score for fatigue during the fatigue-inducing period was located in the medial orbitofrontal cortex (Brodmann's area 10/11) (*P* < .001, uncorrected; Figure 2(a)). In this brain region, adjusted rCBF increased during the fatigue-inducing period in each task session although between the sessions, there was a small reduction in fatigue sensation because of a rest condition (Figure 2(b)). No brain regions exhibited negative correlations in activities with the subjective fatigue sensation.

**Figure 3:**
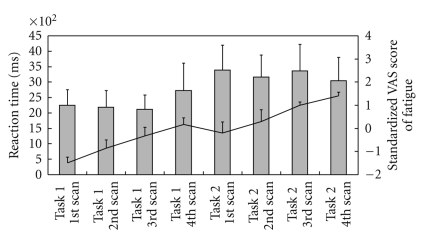
Task performance and standardized visual analogue scale (VAS) score for fatigue during the task trials. The bar graphs show reaction time, and the line graphs show standardized VAS score for fatigue, respectively (mean and SD).

The task performance assessed by mean reaction time of ATMT and the standardized VAS score for fatigue was shown in Figure 3. During the time course of the fatigue-inducing period, although subjective level of fatigue was increased, the task performance was not significantly altered.

## 4. Discussion

During the PET experiment, subjects exhibited a significant increase in subjective score of fatigue sensation, suggesting that they in fact experienced increased sensation of fatigue during the mental tasks and that the tasks used in the PET experiment were fatigue-inducing. As for the subjective sensation of fatigue, we would like to emphasize that we recorded the fatigue VAS score while subjects were performing the fatigue-inducing task trials. This online simultaneous recording of subjective sensation of fatigue enabled us to reliably evaluate the correlation between the fatigue sensation and adjusted rCBF, thereby to identify the brain region associated with subjective fatigue sensation.

We normalized fatigue sensation score in order to minimize between-subject variance in the generalized linear matrix model. Since the aim of our study was to find task-independent brain areas associated with fatigue sensation, pairs of adjusted rCBF value and the normalized fatigue sensation score from multiple subjects were included in one statistical model. The design matrix of the correlation analysis based on this point of view might identify the brain regions related to fatigue sensation independent of types of tasks. It has been shown that was the tendency that the subject had higher level of fatigue sensation in the latter session. Although this design matrix could not eliminate order effect completely, since series of the normalized VAS score were not linearly increased because of the resting condition between Task 1 and 2, the order effect of the fatigue sensation might be minimized.

Orbitofrontal cortex receives inputs from various regions of sensory cortex [17–21]. The medial orbitofrontal cortex may play pivotal roles in terms of feedback from sensory, motor, and cognitive systems [7]. In addition, as referred to as somatic marker hypothesis, medial orbitofrontal cortex is associated with other types of emotion, feeling, and even decision making [22]. Accordingly, the brain region may also play pivotal roles in terms of controlling work output to avoid further fatigue.

We could not dissociate the effects of fatigue from the effects of time. This is a limitation of our study. However, since task performance was not altered during the trials, those effects seem to be minimal.

In conclusion, we have located a brain region associated with fatigue sensation. Today, there are no proper scales for measuring the level of fatigue sensation, and, in the case of fatigue with little or no fatigue sensation, a very serious problem may occur; since fatigue sensation is an indispensable bio-alarm to order rest, little or no fatigue sensation might cause further fatigue and the further fatigue might result in a homeostatic catastrophe causing death, which is known in Japanese as “karoshi.” Therefore, it is important to obtain comprehensive information about fatigue sensation, to develop objective and quantitative evaluation methods of fatigue sensation, and to establish effective treatment strategies for fatigue and its sensation. Our findings provide a new perspective on the neural basis of fatigue.
